# Structural and thermodynamic analysis of factors governing the stability and thermal folding/unfolding of SazCA

**DOI:** 10.1371/journal.pone.0249866

**Published:** 2021-04-15

**Authors:** Shashi Kumar, Parag A. Deshpande

**Affiliations:** Quantum and Molecular Engineering Laboratory, Department of Chemical Engineering, Indian Institute of Technology Kharagpur, Kharagpur, India; University of Akron, UNITED STATES

## Abstract

Molecular basis of protein stability at different temperatures is a fundamental problem in protein science that is substantially far from being accurately and quantitatively solved as it requires an explicit knowledge of the temperature dependence of folding free energy of amino acid residues. In the present study, we attempted to gain insights into the thermodynamic stability of SazCA and its implications on protein folding/unfolding. We report molecular dynamics simulations of water solvated SazCA in a temperature range of 293-393 K to study the relationship between the thermostability and flexibility. Our structural analysis shows that the protein maintains the highest structural stability at 353 K and the protein conformations are highly flexible at temperatures above 353 K. Larger exposure of hydrophobic surface residues to the solvent medium for conformations beyond 353 K were identified from H-bond analysis. Higher number of secondary structure contents exhibited by SazCA at 353 K corroborated the conformations at 353 K to exhibit the highest thermal stability. The analysis of thermodynamics of protein stability revealed that the conformations that denature at higher melting temperatures tend to have greater maximum thermal stability. Our analysis shows that 353 K conformations have the highest melting temperature, which was found to be close to the experimental optimum temperature. The enhanced protein stability at 353 K due the least value of heat capacity at unfolding suggested an increase in folding. Comparative Gibbs free energy analysis and funnel shaped energy landscape confirmed a transition in folding/unfolding pathway of SazCA at 353 K.

## 1 Introduction

Carbonic anhydrases (CAs) constitute a family of Zn-containing metalloenzymes which catalyze the reversible hydration of CO_2_ to bicarbonates and protons [1–4], as shown in Eq (1).

$$\begin{array}{c} {\text{CO}}_{2}+{\text{H}}_{2}\text{O}⇌{\text{HCO}}_{3}^{-}+{\text{H}}^{+} \end{array}$$ (1)

The enzymes of this family are known to exhibit one of the fastest reaction rates known so far in nature [1]. CAs are ubiquitously found in bacteria, algae, plants as well as in eukaryotic animals. These are enconded by six evolutionarily distinct gene families *viz*., *α*, *β*, *γ*, *δ*, *ζ*, and *η* [5, 6]. These different classes of CAs have different structural folds and share low sequence homology [7, 8]. *α*-CAs are the most studied CAs, in which Zn^2+^ ions are present as centres in their active sites, coordinated to three histidines residues [9–11]. In humans, CAs are vital for physiological processes such as regulation of blood pH, regulation of pressure of retinal fluids, and nourishment during bone growth [12]. Recently, CAs have been reported as potential bio-catalysts for industrial applications in carbon capture, storage and sequestration (CCUS) [13]. These enzymes are also of interest as biocatalytic components in biomedical devices such as biosensors and artificial lungs [14]. Unfortunately, the use of CAs in these applications is severely limited by their poor thermal stability [15]. More robust and stable CAs are expected to benefit these applications. Thus, the employment of enzymes extracted from microorganisms living at high temperature range of 323-363 K (*i.e. thermophiles*) can be effectively and efficiently used to overcome these limitations [13]. Recently, several investigators have focused on the development of thermostable CAs to achieve a synergism of their thermostability and high enzymatic activity [16]. In the past, the major focus of the scientific community was dedicated to human CA II (hCAII) isoform to understand its structural and functional features [17]. In this study, we have focussed our attention on another *α*-CA of same CA family, namely SazCA, because of its potential in high temperature biotechnological CCUS applications. We describe the rationale behind the selection of SazCA in the text to follow.

**Fig 1:**
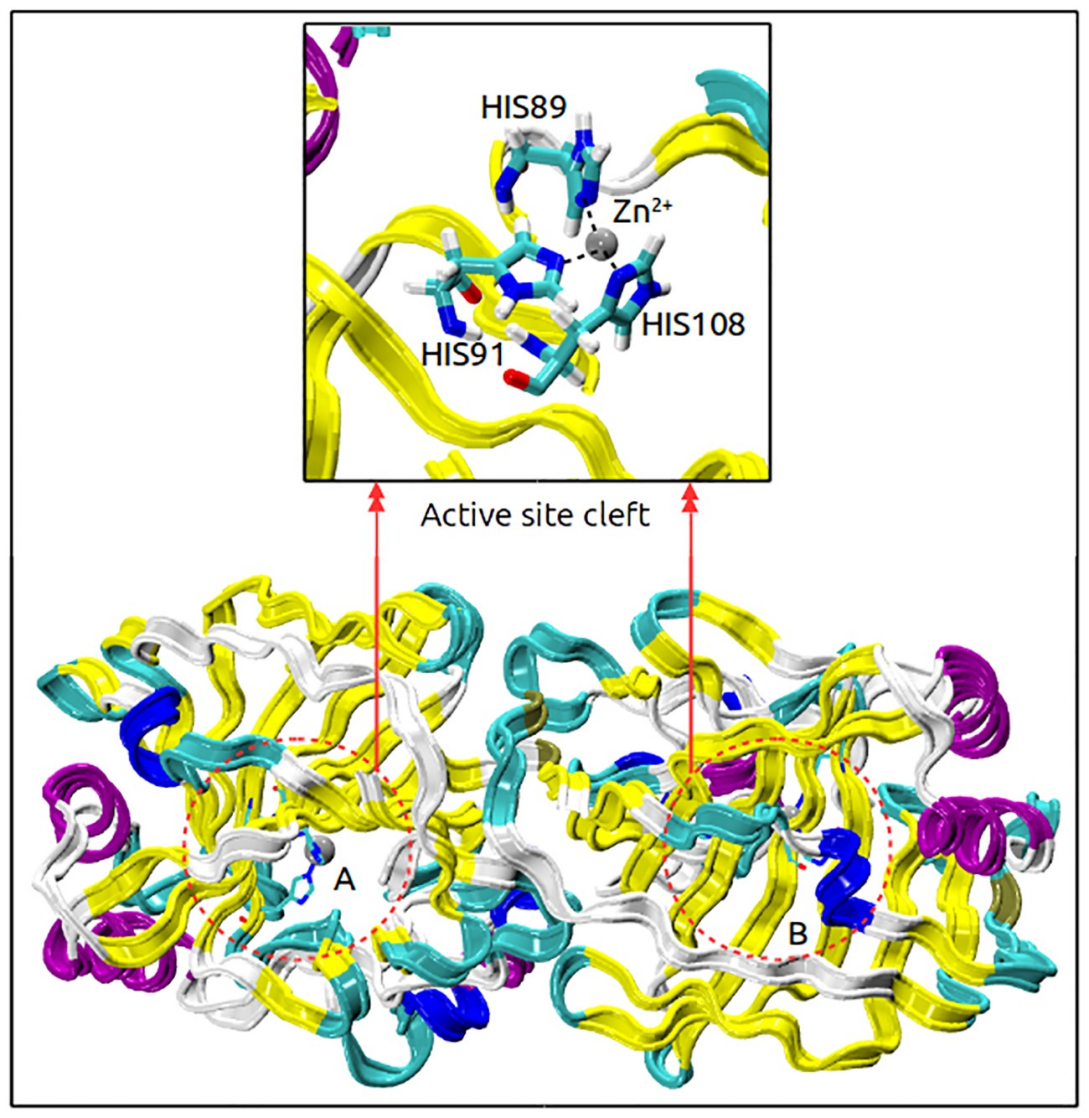
Dimeric structure of thermostable SazCA. Structural representation of dimeric SazCA analysed in this study. The dimer is shown with two chains A, B in Ribbon representation. Zn^2+^ ions are shown as grey spheres and histidine residues involved with direct interactions (active site cleft) with Zn^2+^ are shown in Licorice representation. *α*-helices are shown with violet color, 3_10_-helices with blue color, *β*-sheets with yellow color, loops (turns and coils) are shown in white and cyan respectively.

SazCA is a thermostable CA extracted from *Sulfurihydrogenibium azorense*, a thermophilic bacterium found in hot springs of Azores. It is a dimeric protein with Zn^2+^ active center, as shown in Fig 1. It is 2.3 times more efficient than hCAII for catalyzing the reversible hydration of CO_2_ [18, 19]. The kinetic parameters (k_*cat*_ = 4.40×10^6^ s^−1^; k_*cat*_/K_*M*_ = 3.5×10 ^8^ M^−1^ s^−1^ at 293 K and pH 7.5) for the reaction catalyzed by SazCA show this enzyme to be the most active one among all CAs to date. The thermostability and activity studies have shown this enzyme to be active in a temperature range of 273 to 373 K, with an optimal working temperature for the catalytic activity of about 353 K [19]. SaCA has evolved as a promising bio-catalyst for CCUS because of its exceptional thermostability and owing to the fact that it is the most active *α*-CA known till date for CO_2_ hydration [20]. However, limited information is available on its biophysical behaviour. The folding/unfolding pathways of SazCA are not well explored. Comprehensive data need to be produced on the structure and dynamics of folded and unfolded intermediates of this protein so that a thorough understanding of folding/unfolding pathways of SazCA can be made to provide insights into this class of enzymes which can set a basis for mutations in hCAII or other class of enzymes to improve their stability and/or activity. It is also likely to improve our understanding of protein aggregation and misfolding in general. With this in consideration, we report a molecular dynamics (MD) study of thermal denaturation of SazCA in the present study. We have investigated the effect of temperature on the folding/unfolding pathways of SazCA. We report MD simulations in a range of 293-393 K to unravel the relationship of conformational stability and flexibility in this protein.

Several investigators have studied folding/unfolding characterstics of hCAII under moderate denaturation conditions in the past [21, 22]. Also, studies on unfolding pathways of bovine CA and CAIX in association with conformational stability have been reported [23]. Such details on thermostable SazCA are missing and we provide them by quantifying the secondary structure descriptors, H-bonding descriptors, root mean square deviation (RMSD), root mean square fluctuations (RMSD), radius of gyration (R_*G*)_, percentage of secondary structure contents, solvent accessible surface area (SASA) and pair correlation function (RDF) in this study. Thermodynamic analysis can aid the understanding of reasons behind protein stability and thermal denaturation [24–26]. Hence, thermodynamic analysis of protein stability and pathways of folding/unfolding was done to assess the thermal stability of SazCA. This comprehensive study on the thermal denaturation of SazCA is expected to provide a molecular basis for folded and unfolded ensembles of SazCA, leading to the understanding of structure-function relationships in this protein.

## 2 Results and discussion

### 2.1 Structural analysis

Hydrogen bonding governs the protein secondary structure, and is responsible for its structural stability. There are two types of H-bonds in a protein *viz*., backbone-backbone (B-B) H-bonds and backbone-water (B-W) H-bonds. In this study, we determined both type of H-bonds to probe protein rigidity, thermostablity and unfolding of SazCA, as detailed in the text to follow.

#### 2.1.1 Backbone-backbone H-bonds

**Fig 2:**
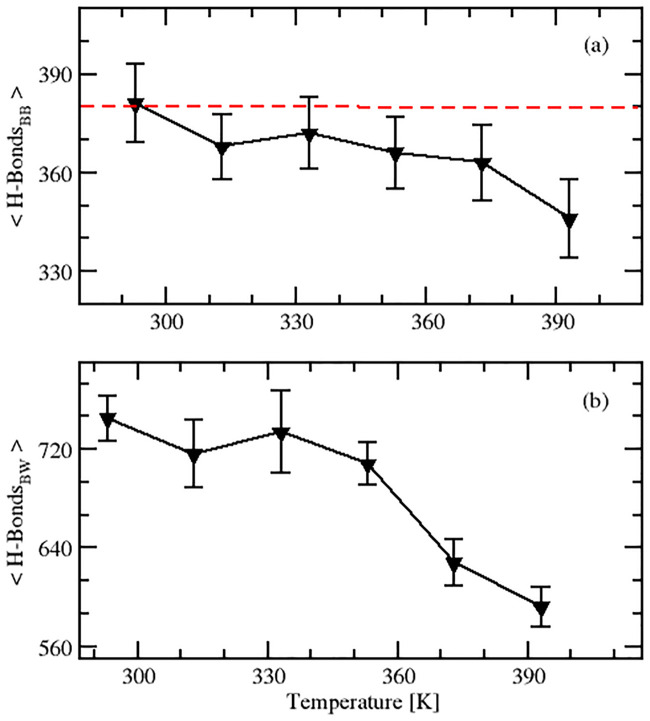
Average number of backbone-backbone and backbone-water H-bonds in SazCA. Analysis of effect of temperature on the number of backbone-backbone and backbone-water H-bonds in SazCA. The variation of average number of backbone-backbone H-bonds with temperature is shown in (a) and the variation of number of backbone-water H-bonds with temperature is shown in (b). The dashed red line in plot (a) represents the number of H-bonds present in the protein crystal structure.

B-B H-bonds are responsible for preserving protein rigidity which in turn helps in improving its thermostability. With an increase in temperature, proteins become unstable due to a decrease in the strength and number of H-bonds. The lesser the number of H-bonds, the higher the flexibility of the protein [27]. The average numbers of B-B H-bonds in well-equilibrated ensembles of the thermophilic SazCA obtained in this study were 381, 368, 372, 366, 363 and 346 at 293, 313, 333, 353, 373 and 393 K, respectively, which indicated a non-monotonous change in the number of B-B H-bonds in SazCA with an increase in temperature. The evolution of the average number of B-B H-bonds as a function of temperature is shown in Fig 2(a). The protein crystal structure had 380 H-bonds, as has been indicated by a red dashed line in Fig 2(a). The number of H-bonds initially decreased with an increase in temperature from 293 to 313 K. This was primarily due to the loss of native conformations in the protein. However, after 313 K, the number of H-bonds increased upto a temperature of 333 K. This increase in H-bonds can be attributed to the high dynamism in the protein which led to the formation of some new bonds at elevated temperatures. Such states were not accessible at low temperatures. A rise in temperature increased the thermal energy of the molecules. Due to the increased thermal energy, the side chains of the protein came closer resulting in interactions leading to the formation of H-bonds. This trend of non-monotonous change in the number of H-bonds with a change in temperature has also been reported by several researchers previously [27–30] for other proteins. Between 333 and 353 K, there was a little change in the average number of H-bonds in the protein. But a sharp decrease in the number of H-bonds was observed between 353 to 393 K. This was due to the fact that the number of B-B H-bonds which were present initially which maintained the conformational stability as well as the thermostability decreased subsequently with an increase in temperature. From Fig 2(a), it was evident that the increase in temperature beyond 353 K induced unfolding in the protein by causing a significant loss in B-I H-bonds which destabilised the native conformations of the protein. The protein maintained its native conformations upto 353 K (optimal working temperature of SazCA). A similar observation of significant loss of B-B H-bonds and a larger exposure of hydrophobic surface residues to the solvent beyond 353 K has been reported to be responsible for unfolding of human isoforms of CA also [23].

#### 2.1.2 Backbone-water H-bonds

B-W H-bonds are responsible for supporting protein-solvent interactions [27]. The interaction of the protein core with the solvent improves when the hydrophobic region of the protein starts unwrapping from its native (wild) folded state to the unfolded state. Larger exposure of hydrophobic surface residues enhances the affinity of protein core to the solvent molecules. The average numbers of B-W H-bonds in SazCA were 745, 716, 734, 708, 628, 592 at 293, 313, 333, 353, 373 and 393 K, respectively. This was similar to the trend for B-B H-bonds. The B-W H-bonds for SazCA first decreased from 293 to 313 K and thereafter increased upto 333 K. The reason behind this behaviour has been explained in the text above. With an increase in temperature from 333 to 353 K, number of H-bonds descended from 734 to 708. With a rise in temperature beyond 353 K, there was a sharp decline in the number of H-bonds as shown in the Fig 2(b). At high temperatures, when the protein unwrapped from its native folded state, the number of B-W H-bonds started decreasing rapidly, as has also been observed by others [27]. The number of B-W H-bonds in the thermophilic SazCA changed only slightly in a temperature range of 293 to 353 K as can be seen in the Fig 2(b) while in the temperature range of 353 to 393 K, the number of native conformations altered significantly due to a remarkable decreased in the number of H-bonds. This indicated that protein was stable upto 353 K and after that unfolding initiated.

#### 2.1.3 Secondary structure assignments

Change in the secondary structure of a protein is an important indicator of the folding/unfolding process. To explore folding/unfolding of SazCA, we calculated the percentage amount of each secondary structure in SazCA as a function of temperature. The comparative secondary structure analysis of SazCA at different temperatures is shown in Table 1, where B, E, T, G, I, H, C represent the percentage of isolated *β*-sheets, extended *β*-sheets, turns, 3_10_-helices, *π*-helices, *α*-helices and coils, respectively. It can be observed that between 293 and 353 K, there was a significant increase in the percentage amounts of *α*-helices (including 3_10_-helices) and *β*-sheets (including isolated *β*-sheets). The corresponding decrease in the amount of turns and coils can also be observed. At 293 K, the percentage amount of helices (*α*-helices and 3_10_-helices) was 6.5%. With a rise in temperature, an increase in the percentage amount of helices was observed, and these were 9.9% at 353 K. Similarly, the percentage amount of *β*-sheets (extended *β*-sheets, isolated *β*-sheets) was 35.5% at 293 K and 37.2% at 353 K. On comparing the helices and sheets, it was observed that there was a small (approximately 2%) increase in the *α*-helices and *β*-pleated sheets from 293 to 353 K. The increase in the percentage amount of helices and sheets suggested higher structural rigidity and a decrease in the flexibility of the protein. A possible reason behind an increase in percentage of helices and sheets was that with an increase in temperature, protein became more flexible gaining a higher access to H-bonds. The secondary structures of the protein, however, lost their integrity with a further increase in temperature. With an increase in temperature, due to the high rate of dynamism in the protein, formation of new interactions took place leading to the formation of stronger bonds [29, 30] upto moderate temperatures. The highest percentage amount of helices and sheets were found at 353 K. The percentage contributions from *α*-helices and *β*-pleated sheets had the highest impact on the structure of the protein at 353 K resulting in retaining the maximum amount of secondary structure assignments. This contribution was not found for rest of the conformations at 373 and 393 K, which led to significant alterations in the secondary structures at these temperatures. This decrease in the percentage amount of helices and sheets can be observed in the Table 1. An increase in the formation of secondary structure assignments induced folding and subsequently unfolding with a decrease in helical and sheets contents. Experimental secondary structure assignments data obtained from the crystal structure of SazCA have also been presented in Table 1 for comparison. 353 K conformation was found to be in good agreement with available experimental data. The highest percentage of secondary structure contents found at 353 K configuration suggested that the structure was in its stable folded state and unfolding started after this temperature.

**Table 1:** Percentage contribution of secondary structure contents in SazCA as a function of temperature obtained from MD simulations, where B, E, T, G, I, H, C represent the amounts of isolated *β*-sheets, extended conformations (*β*-sheets), turns, 3_10_-helices, *π*-helices, *α*-helices and coils, respectively. The secondary structure assignment data of crystal structure (experimental) of SazCA have also been shown for comparison.

Temperature (K)	B (%)	E (%)	T (%)	G (%)	I (%)	H (%)	C(%)
293	0.7	34.8	30.1	1.3	0	5.2	27.9
313	0.9	35.3	29.4	2.3	0	5.4	26.5
333	1.0	35.7	28.7	2.6	0	5.5	26.5
**353**	**1.3**	**35.9**	**27.2**	**3.9**	**0**	**6.0**	**25.6**
373	0.9	34.6	29.4	2.8	0	4.4	27.9
393	0.7	33.9	30.3	1.3	0	4.0	29.8
**Experimental**	**1.3**	**35.9**	**28.3**	**2.9**	**0**	**5.4**	**26.1**

From the comparison of all sampled configurations with secondary structure data of the experimental conformation of SazCA, it was found that 353 K configuration was associated with the maximum number of native conformations. Our computational findings are in a close agreement with H-bond analysis discussed above in this study as well as with the experimental findings. Therefore, it can be deduced that the structural conformation at 353 K supported a compact structure with the highest thermostability.

**Fig 3:**
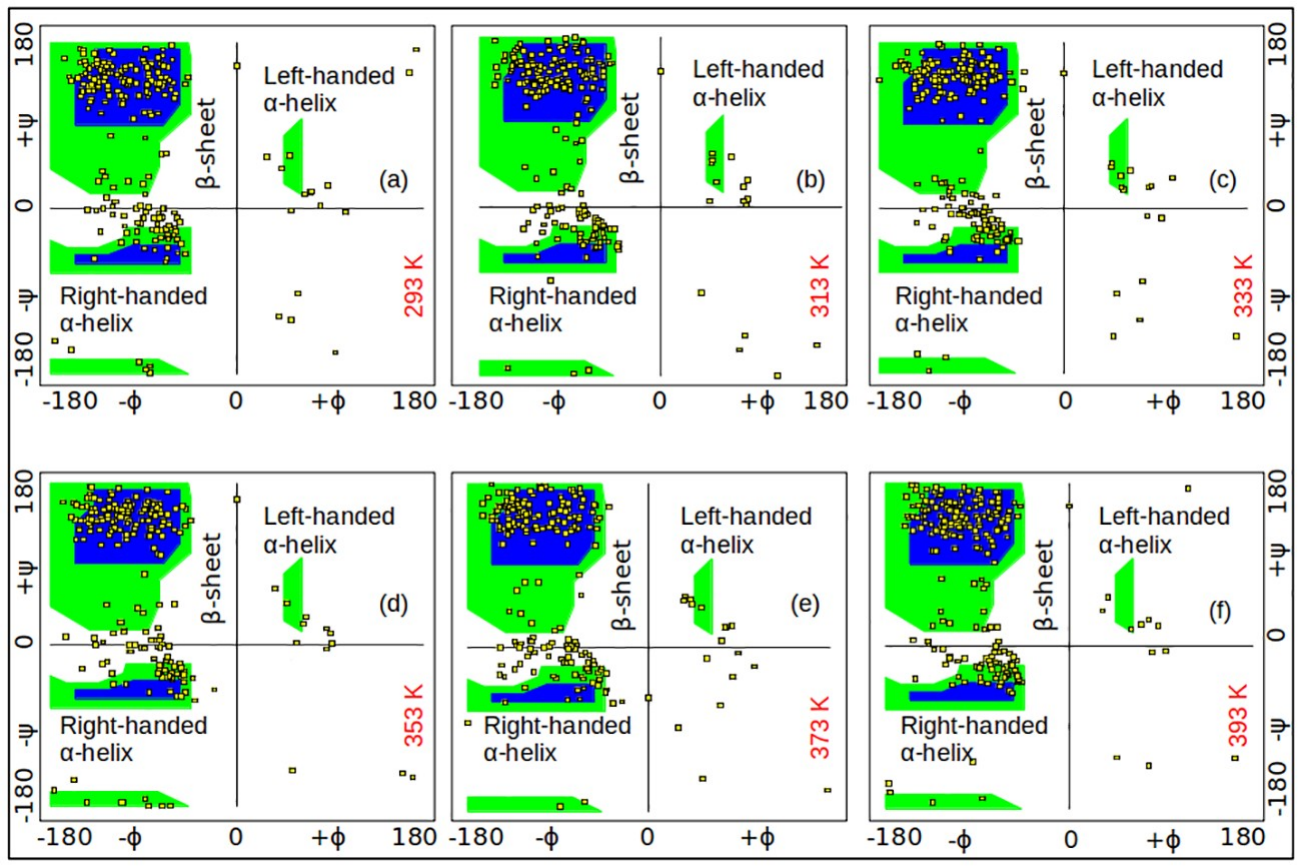
Ramachandran plot analysis. Ramachandran plot of simulated conformational ensembles of SazCA at different temperatures. The blue regions, green regions and white regions represent favoured, allowed and disallowed conformations, respectively. The square symbols represent the simulation data.

Ramachandran plot is an important means to check the quality of the computed 3-D structure of a protein. Ramachandran plot analysis was carried out for well equilibrated conformations of SazCA at different temperatures to analyze the protein structures with energetically allowed conformations. 2-D Ramachandran plots of various conformations of SazCA at different temperatures are shown in Fig 3. The blue regions indicate the most populated and favoured conformations, the green regions represent the allowed conformations, while the white regions correspond to disallowed conformations. Quadrant I shows a region where some conformations are allowed, corresponding to left-handed *α*-helices. Quadrant II shows the largest region in the Ramachandran plot where the most favoured conformations of proteins lie and it corresponds to the *β*-strands. The III quadrant corresponds to the right-handed *α*-helices and it is the second largest region in the Ramachandran plot. The IV quadrant has no specific outlined region and it contains the most disallowed conformations. From Fig 3, it is clear that there was a noticeable difference in the amount of residues present in the most favoured and allowed regions. On comparing, it was found that most of the residues corresponding to 353 K configuration were located in the most favoured region as shown in Fig 3(d). Also, less number of residues were present in the disallowed region at this temperature. A possible reason behind this is lesser steric hindrance among the residues for SazCA at 353 K. We quantified the trends shown in the Ramachandran plot of Fig 3 to get a clearer idea of the changes in the population of the conformations in favoured and disallowed regions as a function of temperature. The quantification has been given in Table 2. It can be seen that the percentage of favoured regions go through a maxima with the highest percentage of allowed regions observed at 353 K. A close correspondance was observed for the population of the conformations in disallowed regions with the minimum population of disallowed conformations at 353 K. This supported our observation that the protein exhibited the most stable geometry at 353 K. The findings of Ramachandran plots were also in a good agreement with the quantitative analysis of secondary structure assignments where maximum number of helices and sheets were observed for this conformation. Therefore, it can be concluded that the system geometry at 353 K was the most stable.

**Table 2:** Percentage occurence of residues in favoured and disallowed regions of SazCA, as obtained from the Ramachandran plot at various investigated temperatures.

Temperature (K)	Favoured region	Disallowed region
293	92.13	2.02
313	92.13	1.37
333	92.36	1.34
353	94.60	1.31
373	91.68	1.62
393	91.68	1.79

#### 2.1.4 Analysis of the solvation shell

Proteins perform their functions in an aqueous environment [31]. In general, water around proteins can be divided into three categories: (1) the bulk water that surrounds the protein, (2) the bound water that forms H-bonds, and (3) the hydration water at the protein surface which has direct interactions with the protein [32–35]. Protein-bound water (*i.e*. bound water and hydration water) form strong interactions with proteins. Protein-bound water molecules are essential to the structure and functions of the protein and they also affect the local protein structure as they contribute to several properties of the protein such as protein stability, protein folding, drug docking and oligomeric formation [36]. Therefore, in order to describe the structure-dynamics of solvent layer around the protein surface, radial distribution function (RDF) of the protein backbone C_*α*_ atoms with respect to the water oxygen was determined. The dynamics of water molecules at the protein interface in terms of probability distribution and water mobility for the simulated frames were analyzed using the following equation [37]:

$$\begin{array}{c} g\left(r\right)=\frac{V}{4\pi N_{f}N\left(N-1\right)r^{2}\delta r}\sum_{n=frames}\sum_{i}\sum_{j\neq i}\delta \left(r-r_{ij}\right) \end{array}$$ (2)

where *N(N-1)/V* = number of atoms pairs per unit volume, *4π*r^2^*δr* = volume sampled at distance *r*.

**Fig 4:**
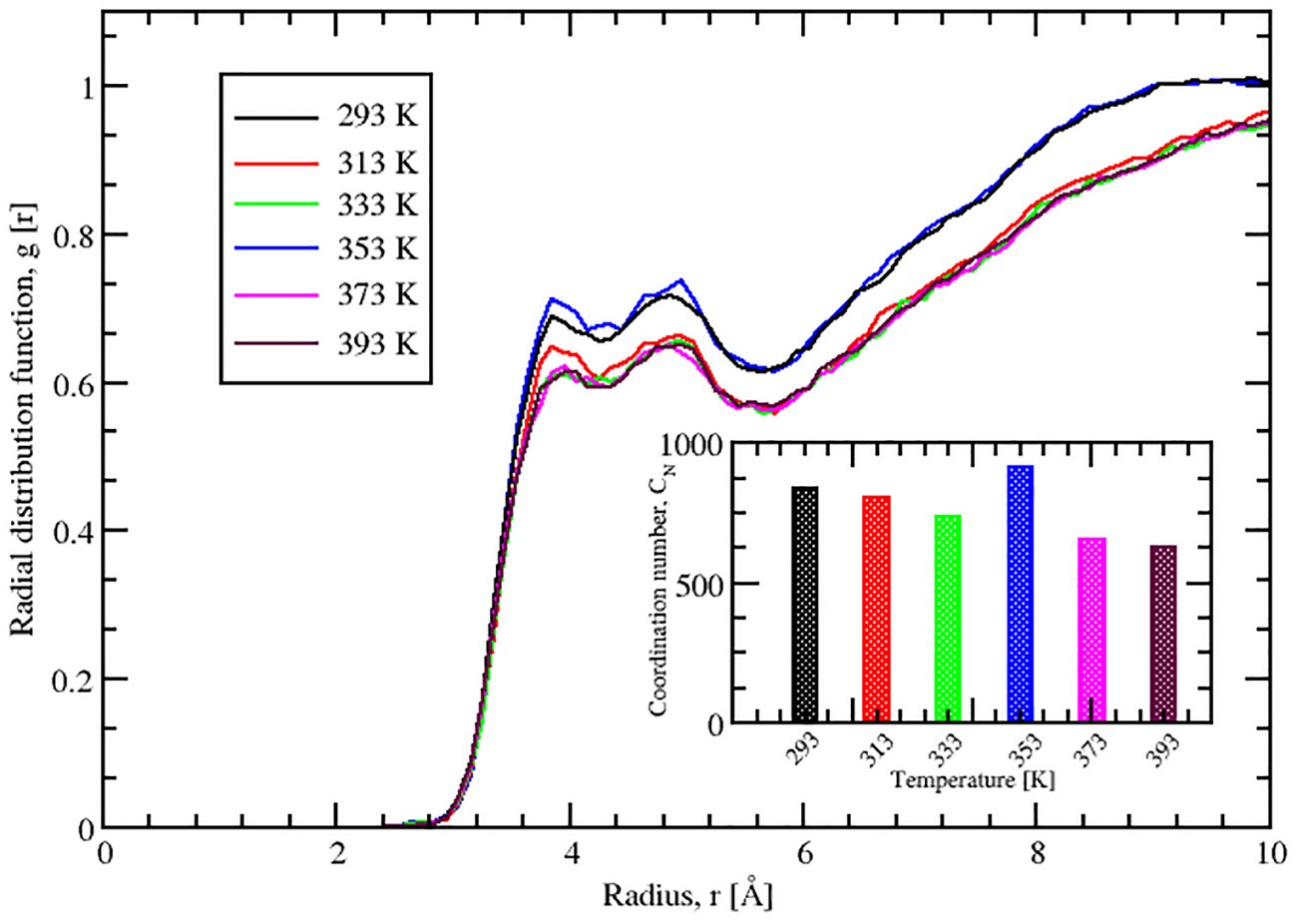
Radial distribution function analysis. Radial distribution function of the mass center of solvent molecules with respect to the protein surface at different temperatures. The variation of the coordination number of water molecules around the protein in the first solvation sphere with temperature is shown in the inset.

We calculated the RDFs for SazCA at the aforementioned temperatures *viz*., 293, 313, 333, 353, 373 and 393 K. The distribution of the solvent layers around the protein surface is indicated by the RDF shown in Fig 4. The calculated RDFs showed that there were two maxima representing two hydration shells around protein surface, at a radial distances of 3.85 Å and 4.95 Å, respectively. The first hydration shell consisted of solvent molecules interacting with the charged or polar species of the protein whereas the second shell indicated the presence of van der Waals interactions between the protein and water molecules [38]. A comparison of the RDFs at investigated temperatures showed that there were negligible differences in the first peak positions but significant differences in their amplitudes. The highest intensity peak in the first solvation shell can be observed for SazCA at 353 K compared to those at the rest of the temperatures. The reason behind this high intensity peak at 353 K was the high local structuring of water molecules around the protein surface. These local ordering of water molecules in the vicinity of protein surface was due to the steric hindrance and specific interactions between the protein and water molecules [38]. It also signified the high ordering of solvent molecules with protein backbones atoms through strong hydrophilic interactions. Also, on comparing the second solvation shell, a sharp and high intensity peak was observed at a temperature of 353 K but relatively at a larger distance compared to the peak in the first solvation shell. It suggested the existance of ordered water molecules near the protein surface. After the second solvation shell, a further increase in *g*(*r*) extended the peaks close to 1 at *g*(*r*) = 10, indicating the hydration water to have reached to the bulk properties. The RDF of SazCA at 353 K was sharper indicating that protein bound water was more ordered and less mobile than the bulk water. Therefore, it can be concluded that at 353 K, SazCA had a better and stable representative structural ensemble elucidating the structural stability, folding and dynamical properties compared to structural ensembles at other temperatures. The variation of coordination number of water molecules around the protein in the first solvation sphere with temperature was quantified. The variation is shown in the inset of Fig 4. It can be seen that the highest coordination number was observed at 353 K showing the maximum ordering of the water molecules around the protein resulting in its stabilisation at 353 K.

#### 2.1.5 Further structural details

In order to develop further insights into the conformational stability and flexibility in SazCA, we did a set of analyses such as RMSD, RMSF, R_*G*_ and SASA of protein to figure out the deviations of the protein from the crystal structure. Simulations at elevated temperatures showed that SazCA was stable in a temperature range of 293 to 353 K, indicated by the low RMSD observed between the simulated conformations and initial crystal structure. On the other hand, SazCA displayed a sharp increase in RMSD from 353 to 393 K, suggesting the onset of an increase in fluctuations and denaturation of this enzymes at high temperatures [27]. The average values of RMSD obtained at different configurations as a function of temperature are shown in the S1 Table in S1 File. RMSF analysis also revealed fluctuations of lower magnitude in a temperature range of 293 to 353 K. It suggested lesser structural flexibility at this temperature range. Beyond this temperature, there was a significant decrease in the structural rigidity and an increase in the flexibility. The associated residues responsible for higher RMSF at elevated temperatures were VAL98, ASN99, GLY100, LYS101, GLU145 and HIS207. [39] These highly flexible amino acid residues and their variation in length with simulation temperature are shown in S2 Table in S1 File. These residues were identified for the enhanced flexibility and unfolding of conformational ensembles of SazCA at higher temperatures. Further analysis of R_*G*_ indicated a gradual increase in the magnitude of R_*G*_ form 293 to 353 K, suggesting partial expansion in the protein structure. A large increase in the value of R_*G*_ at 373 and 393 K, suggested larger expansion in the protein structure. The breaking of the native contacts [23] at higher temperature can be a possible reason behind the expansion of the protein. This can be correlated with the analysis of secondary structure assignments described above in this study. The average R_*G*_ of SazCA at different temperatures are shown in S1 Table in S1 File. The complete analysis of MD simulations involving the structural parameters (RMSD, RMSF and R_*G*_) with an increase in temperature can be find out in our earlier investigations on this enzyme [39].

To further develop an understanding of the structural stability, we performed SASA analysis at different investigated temperatures. SASA is considered to be a crucial parameter in the determination of stability of proteins [40]. We examined the SASA of all the sampled conformations and the variation of SASA of each sampled conformations as a function of simulation temperature is shown in S3 Fig in S1 File. From the analysis of S3 Fig in S1 File, it is clear that there were significant changes in the magnitudes of SASA obtained at different temperatures. The average SASA obtained for 293, 313, 333, 353, 373 and 393 K configurations were 1184 × 10^2^ ± 6.45 Å^2^, 1147 × 10^2^ ± 3.26 Å^2^, 1129 × 10^2^ ± 3.11 Å^2^, 1104 × 10^2^ ± 2.72 Å^2^, 1154 × 10^2^ ± 3.28 Å^2^ and 1189 × 10^2^ ± 5.14 Å^2^, respectively. From the comparison of average SASA, we found that least SASA was associated with 353 K configuration. Our reported results of SASA were consistent with the analysis of secondary structure assignments, hydrogen bonding pattern and radius of gyration. Lesser SASA obtained for 353 K configuration inferred less structural disruption during the simulations runs. It can also be deduced that the amino acid residues were intact inside the protein core and less surface area was exposed to solvent medium. A possible reason was higher folding [41] for this conformation as we have discussed in the analysis of unfolding pathways. From SASA analysis of 353 K configuration, it again appeared to be the best conformation for the structural stability due to the high folding and less initial drift from the native structure.

### 2.2 Thermodynamic analysis of unfolding pathways

**Fig 5:**
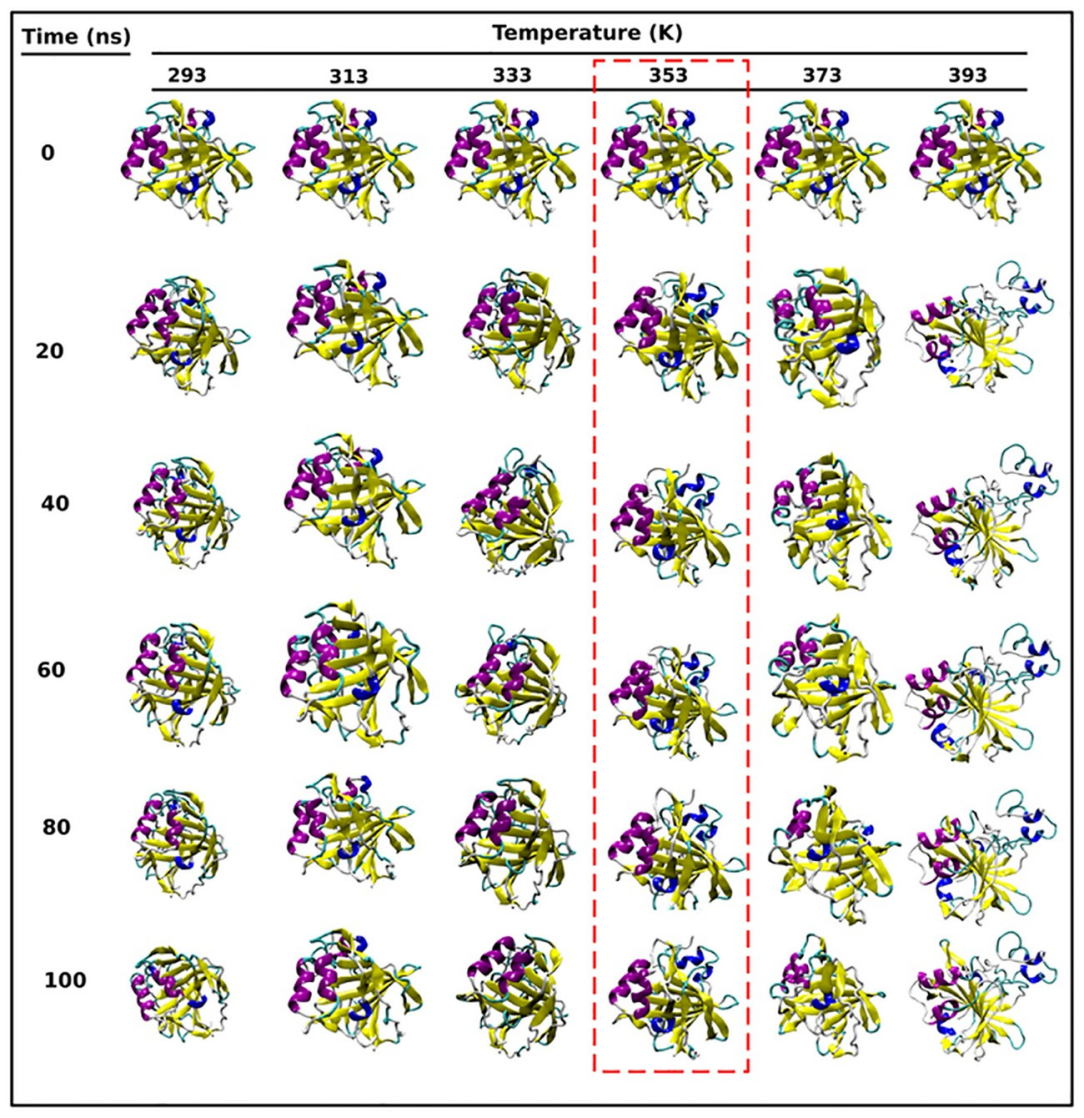
Unfolding pathways of SazCA. The structural snapshots taken at 0, 20, 40, 60, 80 and 100 ns of different configurations of SazCA at 293, 313, 333, 353, 373 and 393 K, describing the unfolding pathways with respect to its native state. *α*-helices are shown with violet color, 3_10_-helices with blue color, *β*-sheets with yellow color, loops (turns and coils), are shown in white and cyan color. The native-like structure found at 353 K is represented with red color dashed rectangle.

Various analyses detailed till now all point out to the fact that the protein exhibited the highest stability at 353 K and hence, thermal denaturation of the protein is expected afterwards with an increase in temperature. We carried out the thermodynamic analysis of the unfolding process, described in the text to follow. We firstly analysed the pathway followed by the protein during its unfolding. Structural snapshots of all the sampled conformations of SazCA throughout the MD trajectories at 0, 20, 40, 60, 80 and 100 ns have been presented in Fig 5. There were no significant changes in *α*-helix contents at 293 K but a significant increase in the *β*-sheets was observed between 60 to 100 ns. An increase in the amount of *α*-helices and *β*-sheets were observed at 313 and 333 K. Also the increase in size of *α*-helices and *β*-sheets can be observed between 20 to 100 ns. At 353 K, there was a significant increase in the secondary structure contents compared to its initial conformation, especially in 3_10_-helices. Also, an increase in the sizes of the *β*-pleated sheets and *α*-helices were seen between 20 to 100 ns in 353 K configuration. A subsequent decrease in the amount of turns and coils can also be observed. The maximum amount of secondary structures found at 353 K conformation suggested lesser flexibility and higher thermal stability of the protein structure at this temperature. The other reason associated with the higher thermostability and lesser flexibility of 353 K configuration was strong salt bridge networks and interactions between the residues, as described elsewhere [39]. These strong salt bridges networks were responsible for increased amount of helices and sheets in SazCA at 353 K. After 353 K, there were structural distortions at 373 and 393 K. Significant losses in *α*-helices and *β*-sheets were observed at both the temperatures. The protein was intact at 353 K but unfolding prevailed after 353 K, which resulted into lesser contents of helices and sheets and more number of turns and coils at 373 and 393 K. This phenomena of unfolding can be seen in Fig 5. High flexibility and compromised thermostability at elevated temperatures (373 and 393 K) were due to five unstable salt bridges (ASP113-LYS81, ASP115-LYS81, ASP115-LYS114, GLU144-LYS143 and GLU144-LYS206) [39]. Our findings of unfolding pathways were in agreement with the quantitative analysis of secondary structures analysis reported above in this study. Hence, it can be inferred that the structural conformations at 353 K were the most thermostable conformations with their higher resemblance to the native state and the association of higher contents of secondary structure assignments.

There exist close relationships among the protein stability, structure, function, and the associated energetics. Proteins undergo conformational changes during their functional performance, resulting in changes in properties which are governed by the energetics. The contributions of stability interactions to the protein structure, which are defined by the structure, are known to be temperature dependent, where some interactions may be more dominant than others at high temperatures and less dominant than others at low temperatures or *vice versa* [42–45]. The energetics not only determines the stability of the structure but also identifies the domains of protein which undergo large conformational changes with increasing temperatures when compared to the native state of the protein. Therefore, in order to get a better understanding of the thermal denaturation of SazCA, we carried out a macroscopic description of the protein stability in terms of thermodynamic parameters, as described in detail in the sections to follow.

#### 2.2.1 Prediction of folding free energy change

**Fig 6:**
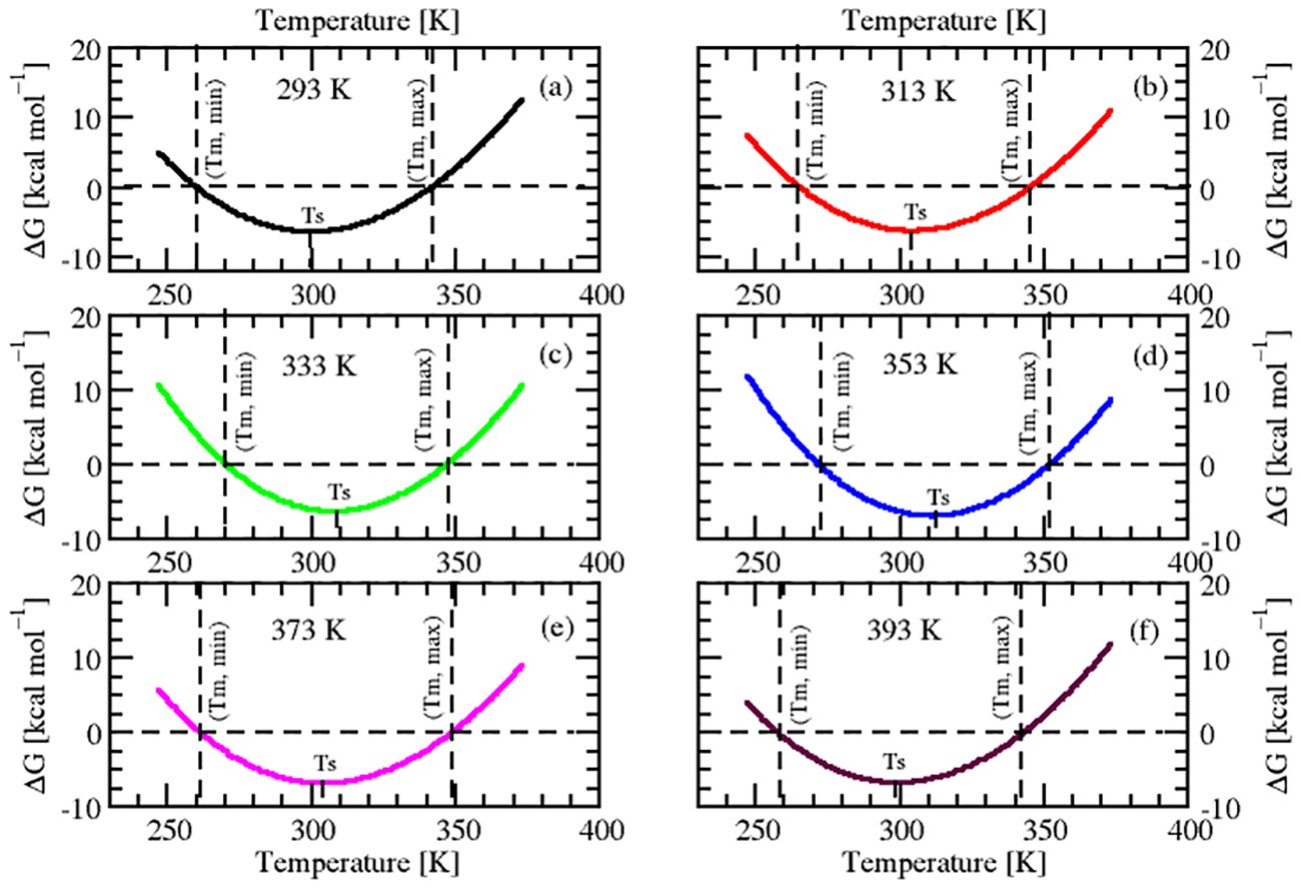
Stability curves of SazCA. Stability curves of different conformational ensembles of SazCA illustrating the temperature dependence of the folding free energy ΔG. Temperatures of cold unfolding T_*m*,*min*_, hot unfolding T_*m*,*max*_ and temperature of maximum stability T_*s*_ have been indicated in the plots.

Thermodynamic stability of a protein at a given temperature is governed by the folding free energy change ΔG(T) at that temperature and the thermal stability via the melting (denaturation) temperature T_*m*_. Knowledge of ΔG is important because all the other thermodynamic parameters that characterize the protein folding transition can be extracted from it. The protein folding transitions (stability curves) can be described by the Gibbs-Helmholtz equation [46, 47]:

$$\begin{array}{c} \Delta G\left(T\right)=\Delta H_{m}\left(1-\frac{T}{T_{m}}\right)-\Delta C_{P}\left(T_{m}-T+T\ln\left[\frac{T}{T_{m}}\right]\right) \end{array}$$ (3)

where T_*m*_ is the melting temperature, which is the midpoint of the thermal denaturation, ΔH_*m*_ is the enthalpy of unfolding measured at T_*m*_, and ΔC_*P*_ is the heat capacity change measured at unfolding. We determined the free energy change for all the conformational ensembles of SazCA in a temperature range of 293-393 K. The individual stability curves of different conformational ensembles of SazCA illustrating the temperature dependence of change in free energy, ΔG, are shown in Fig 6. By convention, ΔG(T), ΔH_*m*_ and ΔC_*P*_ have been defined as the difference between folded (native) and the unfolded (denatured) state and hence, the nature of resulting stability curve is concave up. As the temperature deviates from the maximal stability temperature, the protein can unfold either due hot denaturation or cold denaturation. The temperature corresponding to the maximum stability has been marked by T_*S*_ in Fig 6. The temperatures at the two transitions *i.e*., hot and cold unfolding have been labelled as T_*m*,*max*_ and T_*m*,*min*_ respectively. From Fig 6, it can be observed that there was temperature T_*S*_ at which the protein conformation was in most stable form. As the temperature increased or decreased, ΔG also changed to favour denaturation and protein conformations became more unstable.

**Fig 7:**
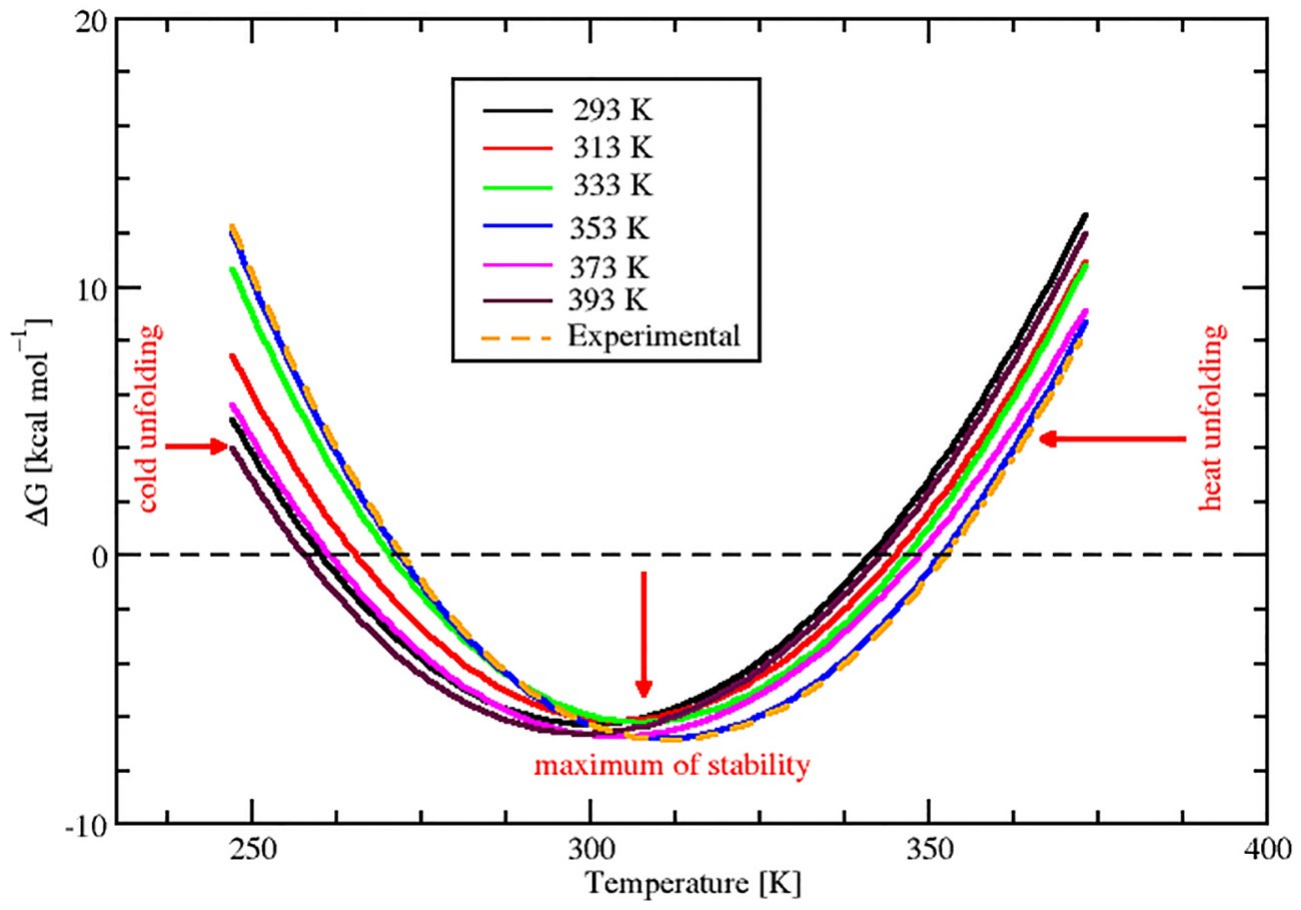
Superimposed stability curves. Comparative stability diagram of predicted Gibbs free energy for different conformational ensembles of SazCA. The experimental Gibbs free energy has also been plotted with dashed line for comparison.

From the comparison of the individual stability curves at different temperatures in Fig 6, it was found that the temperature of maximum stability, T_*S*_, initially underwent a shift towards the high temperature, and then retracted back to lower temperatures with an increase in temperature. For conformational ensembles from temperatures 293 to 353 K, there was a right shift in maximum stability towards high temperature region and after 353 K upto 393 K, it showed a decrease. The maximum shift towards high temperature and maximum stability temperature was found for conformational ensemble at 353 K. This change in maximum stability can be easily visualized in Fig 7 where the stability curves of all the sampled configurations have been superimposed. Also, it was found that there was a global decrease in free energy change as the temperature increased from 293 to 353 K and the least value was observed at 353 K.

**Fig 8:**
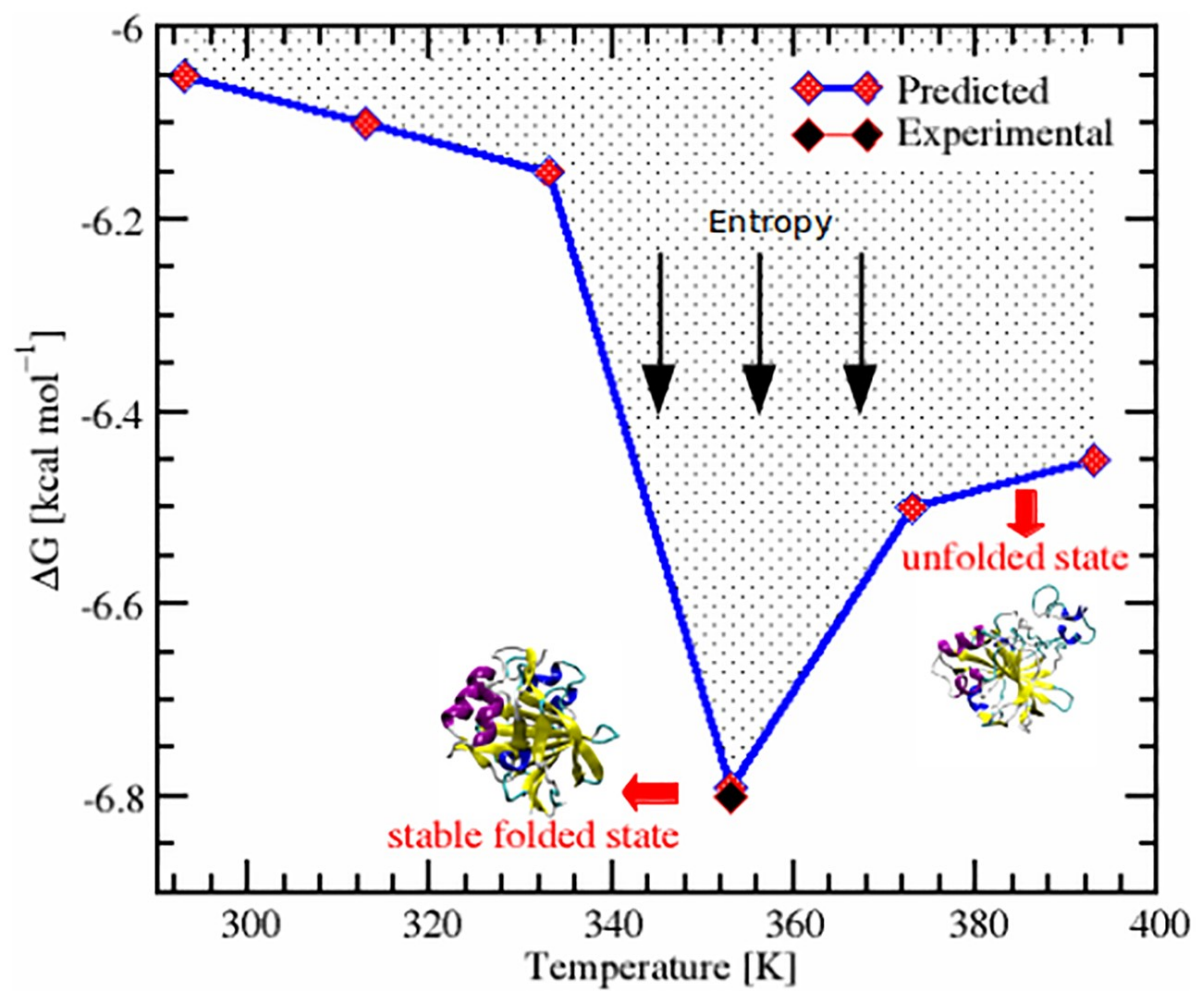
Energy landscape of SazCA. Funnel shaped energy landscape of SazCA representing the relationship of folding/unfolding to the free energy as a function of temperature. The native-like structure found at 353 K is shown in secondary structure representation as the stable folded structure.

The funnel shaped energy landscape of folding/unfolding pathways of SazCA is shown in the Fig 8. It can be observed from the Fig 8 how SazCA approached into its most stable folded (native-like) structure by minimizing the free energy. Unfolding prevailed as temperature rose beyond 353 K. In this canonical depiction of the energy landscape, the depth of the funnel indicated the stability of the protein and width of the funnel represented the conformational entropy of the protein. Hence, these finding suggested 353 K configuration to be a most stable conformation and having the highest resemblance to the native folded state when compared to other tested conformations. Moreover, our findings were in a close agreement with the experimental observations which also showed 353 K to be the temperature with the most stable configuration. The stability curve for experimental conformation has been shown with regular dashed line in Fig 7. Our findings corroborate with the results reported by Pucci *et al*., that the protein conformation which belongs to maximum stability temperature and lowest value of ΔG has the most stable form and exhibits the highest thermostability (thermoresistance) [48]. Hence, 353 K conformations were the conformations with the highest stability.

#### 2.2.2 Prediction of melting temperature T_*m*_

**Fig 9:**
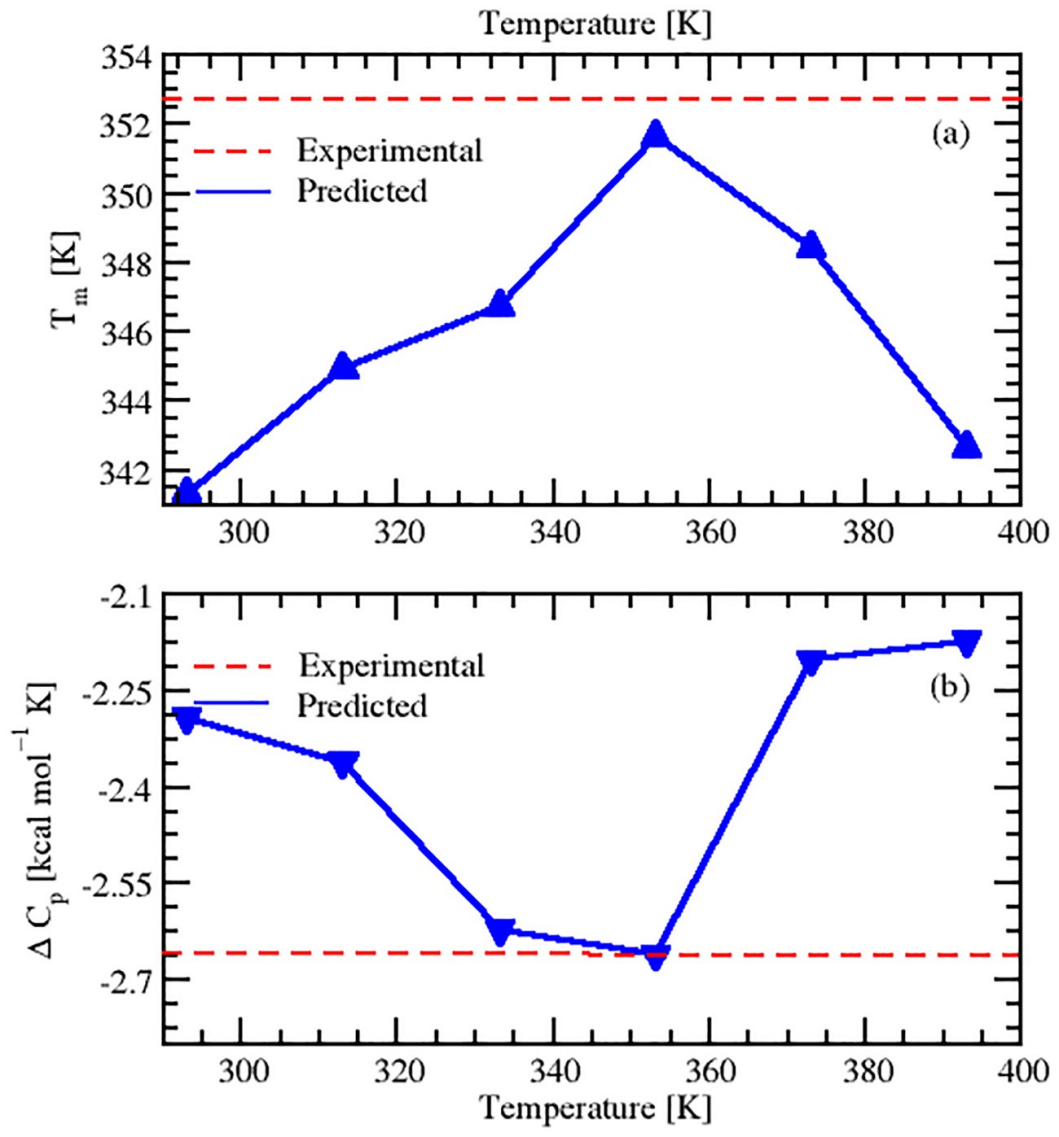
Variation of melting temperatures and heat capacities as a function of temperature. Predicted melting temperatures T_*m*_ (a) and heat capacities (b) at unfolding for different conformations of SazCA. The experimental T_*m*_ and ΔC_*P*_ of the crystal structure have also been plotted for comparison with dashed line.

Apart from the thermodynamic stability in terms of ΔG, the thermal stability in terms of melting temperature, T_*m*_, can be calculated from the stability diagram as an increase in T_*m*_ is associated with free energy of maximum stability. A global decrease in the ΔG implies an increase in T_*m*_. From Figs 6 and 7, it can be observed that there was a significant increase in T_*m*_ of sampled configurations from 293 K to 353 K, but after this temperature, there was a reduction in T_*m*_. It can also be observed that the configuration sampled at 353 K was associated with the highest T_*m*_ (351.65 K) and this temperature (353 K) has been reported to be the experimental optimum temperature. The experimental configuration has a value of T_*m*_ (352.75 K ≃ 353 K) which is close to the our finding of 353 K. Fig 9a shows the variation of T_*m*_ of all sampled conformational ensembles at different temperatures. The corresponding data for thermal stability for different sampled conformations at various temperatures can be found in Table 3. The protein conformations denaturing at higher values of T_*m*_ tend to adopt greater maximum thermal stability [49]. Therefore, our findings suggested 353 K configuration to be the best descriptor for thermal stability because of its association with highest denaturation temperature. It also implied that structural ensemble at 353 K was in a stable folded state and unfolding started after this temperature. A decrease in magnitude of T_*m*_ after 353 K configuration can be observed in Fig 9a and corresponding data in Table 3.

**Table 3:** Predicted and experimental values of thermodynamic and thermal parameters of ensembles of SazCA at different temperatures. The thermal and thermodynamic parameters of crystal structure (experimental) of SazCA are also shown for comparison.

Temperature (K)	ΔH_*m*_ (kcal mol^−1^)	ΔC_*P*_ (kcal mol^−1^ K^−1^)	ΔG (kcal mol^−1^)	T_*m*_ (K)
293	-101.1	-2.29	-6.05	341.35
313	-101.8	-2.36	-6.10	344.95
333	-108.3	-2.62	-6.15	346.75
**353**	**-115.4**	**-2.66**	**-6.79**	**351.65**
373	-103.2	-2.20	-6.50	348.45
393	-102.4	-2.17	-6.45	342.65
**Experimental**	**-116.2**	**-2.66**	**-6.80**	**352.75**

#### 2.2.3 Prediction of heat capacity ΔC_*P*_ at unfolding

To gain more understanding into the thermodynamic stability and to make the analysis more quantitative, we predicted another thermodynamic parameter, heat capacity (ΔC_*P*_) at unfolding. We predicted the ΔC_*P*_ at all investigated temperatures and its variation at different temperatures is shown in Fig 9b. The corresponding data of these ΔC_*P*_ values are shown in Table 3. On comparing ΔC_*P*_, we found a similar trend as we found in the analysis of ΔG. From temperatures 293 to 353 K, there was a decrease in the magnitude of ΔC_*P*_ values followed by subsequent increase from 353 to 393 K. The maximum negative ΔC_*P*_ was found for 353 K conformations. The experimental ΔC_*P*_ has also been plotted and is shown as the regular dashed line in Fig 9b. It was found that our predicted value of ΔC_*P*_ and the experimental were in agreement. From Figs 7 and 9(b), it can be observed that lesser negative value of ΔC_*P*_ at 353 K configuration broadened the stability curve and resulted in an up-shift towards high temperatures. From this, it can be deduced that there was a close relationship between ΔC_*P*_ and T_*m*_ of the protein. The lesser the negative value of ΔC_*P*_, the higher the magnitude of T_*m*_. The protein associated with the less negative value of ΔC_*P*_ had higher denaturing temperature (T_*m*_) and exhibited enhanced thermoresistance [48, 50]. The association of highest melting temperature and least ΔC_*P*_ value at 353 K resulted these conformations to be the most thermoresistant and thermostable. The enhanced protein stability at 353 K suggested an increase in folding [51]. The decrease in melting temperature after 353 K altered the native conformations and suggested a gain in conformational disorder (entropy) due the increase in the disorder of the protein-solvent interactions at higher temperatures. Hence, it can be concluded that at 353 K, SazCA has a transition in the folding/unfolding pathways. The stable folded conformation with highest thermostability was found for 353 K and beyond this temperature, denaturation prevailed.

## 3 Method

The crystal structure of SazCA isolated from *Sulfurihydrogenibium azorense* (PDB ID: 4X5S) [20] was used as the initial guess structure for setting up MD simulations. The protein reported in this PDB had a dimeric arrangement and Zn^2+^ ions were present in each of the monomeric units. The resolution of crystal structure of this PDB of thermostable SazCA was 1.95 Å. The amino acid numbering from the published crystal structure of SazCA was used in this study. Hydrogens were added to the hydrogen-less crystal structure conforming to the following protocol.

SazCA has been reported to be catalytically active for CO_2_ hydration at 293 K and 7.5 pH [19]. Conforming to these conditions, the protonation states of amino acid residues were decided. This effect was characterized by the pK_*a*_ of the amino acid side chain. PSFGEN package [52] was used to add hydrogens to the crystal structure. The protonated protein was then immersed in a periodic cubic water box of dimensions 120 × 120 × 120 Å conforming to the constraint that there was a distance of at least 15 Å between the edge of the water box and the nearest protein fragment. This was done to minimize the interactions of periodic images of the protein. Further, Na^+^ and Cl^−^ counterions were added to the system so as to render the system a zero net charge.

To study the structural and dynamical behaviour of SazCA, MD simulations of the solvated protein were carried out at six different temperatures in a range of 293-393 K, each for a period of 100 ns, with AMBER99SB* [53] force-field using NAMD [54] package. AMBER99SB* was chosen for the protein description following a detailed study by us reported elsewhere [39]. The water model TIP3P [55] was used to solvate the protein as TIP3P water model has been reported to support the sampling of metastable *β*-structures, turns and helical structures [56] which are important in determining the conformational stability of a protein. The steepest descent method [57] of energy minimization was applied for 1000 cycles to minimize the system energy. This was done to eliminate all initial bad stresses and contacts. Long range electrostatic interactions were calculated using Particle Mesh Ewald method and a cut off distance of 12 Å was employed to handle real-space interactions [58]. Once these preliminary steps were completed, the system was allowed to equilibrate using 1 fs time step of integration and 1 bar pressure in isothermal-isobaric (NPT) ensemble. Barendsen’s coupling method was applied to control pressure fluctuations in the system [59]. The SHAKE algorithm was employed to constraint the bonds between the heavy atoms and hydrogens [60]. Isothermal conditions were maintained throughout the MD simulations by Nosé-Hoover’s [61] method where Langevin dynamics [62] was used to control the barostat fluctuations. The results reported in this study are based on well equilibrated final 20 ns simulation trajectories for each system.

The tools provided in the VMD [52] were utilised to analyse different MD trajectories and to render the protein structures. The measurements of the RMSD, RMSF, R_*G*_ and their convergence were computed and the same have been provided in the S1 File. Hydrogen bonding is a key parameter for maintaining protein secondary structure. To calculate H-bonds, we used a geometrical criterion according to which the cutoff distance between the donor and acceptor was less than 3.4 Å and angle was less than 30° for an H-bond to exist [63]. In this study, we considered both intrapeptide as well as interpeptide H-bonds. Analysis of MD trajectories also included the measurement of secondary structure assignments and SASA. STRIDE algorithm was used to measure these parameters. The folding free energies were computed to explore the folding/unfolding pathways of SazCA. SCooP [64, 65] web server was used to calculate the thermodynamic parameters for the stability curves of the protein.

## 4 Conclusions

In the current study, MD simulations were carried out for understanding the molecular basis of thermal stability and folding/unfolding of SazCA. Different stability-determining variables, such as RMSD, RMSF, R_*G*_, SASA, H-bonds, RDF, Gibbs free energy, melting temperatures, secondary structure assignments, unfolding pathways, associated with structural stability and folding/unfolding of the protein were quantified. The effect of elevated temperatures on RMSD, RMSF and R_*G*_ confirmed that the enzyme maintained the structural stability upto 353 K and beyond this temperature, protein conformations were highly flexible. Due to higher resemblance of protein conformations at 353 K to the native state, SazCA exhibited the highest rigidity and thus was the most stable at 353 K. The SASA analysis revealed 353 K conformations to show stable dynamics due the higher rate of folding and less initial drift of the protein structure from the wild structure. An analysis of H-bonds showed that SazCA maintained its native state upto 353 K due the lesser exposure to unfolding for this temperature range. The conformational ensembles of the protein were in the most folded state and unfolding prevailed after 353 K due to a remarkable decrease in the backbone-backbone as well as backbone-water H-bonds and larger exposure of hydrophobic surface residues to the solvent medium. RDF analysis showed 353 K conformations with higher ordering of solvent molecules with the protein backbones atoms through strong hydrophilic interactions to be the stable structural ensemble to depict folding and dynamical properties. Due to the occurrence of higher percentage amount of *α*-helices and *β*-sheets at 353 K, these conformations exhibited the highest thermostability in SazCA.

In addition to providing the insights into the thermal stability of SazCA, we also addressed the effect of temperature on the thermodynamic stability of SazCA. From the thermodynamic stability analysis, it was found that SazCA had the most stable folded conformation at 353 K. The mechanism of maximum stabilisation exhibited by SazCA at 353 K can be summarised into three major outcomes: first, its association with highest T_*s*_ and maximum negative ΔG, second, the highest T_*m*_ which was 351.65 K, close to the experimentally reported optimum working temperature (353 K) of SazCA, and third, the maximum negative ΔC_*P*_ of SazCA at 353 K, which upshifted the stability curve towards high temperature regime. After 353 K, SazCA had a transition in folding/unfolding pathways. Hence, it can be concluded that at 353 K, the enzyme SazCA has most stable folded ensemble with the highest thermostability.

## Supporting information

S1 File (PDF)
