# Supplementary material for: Structural and thermodynamic analysis of factors governing the stability and thermal folding/unfolding of SazCA
Source: PLoS One. 2021 Apr 15;16(4):e0249866. doi: 10.1371/journal.pone.0249866 (PMC8049272; doi:10.1371/journal.pone.0249866)
Supplement: S1 File — (PDF) [file pone.0249866.s001.pdf]

Structural and thermodynamic analysis of factors governing the stability and thermal folding/unfolding of SazCA

Shashi Kumar and Parag A. Deshpande*

Quantum and Molecular Engineering Laboratory, Department of Chemical Engineering, Indian Institute of Technology Kharagpur, Kharagpur 721302, India

*Corresponding author: Email: parag@che.iitkgp.ac.in; Phone: (+91) 3222 283916

1 Structural analysis of SazCA

The molecular dynamics (MD) simulations at 293, 313, 333, 353, 373 and 393 K were performed in order to identify the regions of conformational stability and flexibility. The root mean square deviations (RMSD) was calculated to determine the structural stability using an automated script as implemented in VMD. The deviations were determined with the atomic coordinates of the crystal (native) structure taken as reference. All atom (C, N, O and C_α) and backbones (N, C_α and C) were calculated using the eq. (1).

$$RMSD = \sqrt{\frac{1}{N}\sum_{i=1}^{N}|x_i - y_i|^2} \tag{1}$$

where x_i and y_i are the coordinates of the atom i and the atom in the corresponding reference structure with N being the total number of atoms in the system (N= 94991 in the present case, comprising of 7236 protein atoms and 87755 solvent atoms). The time evolution of average RMSD at different temperatures as simulations progressed, were shown in Figure S1 below. It can be seen from the Figure S1 (293-393 K) that this structural parameter showed a convergence at all temperatures and the results reported by us in the present study at the end of complete 100 ns MD simulation run represented a well-equilibrated system. The variation of average values of RMSD at different temperatures were shown in the Table S1.

To identify the structural flexibility of the protein (C_α only), root mean square fluctuations (RMSF) were computed using the eq. (2). RMSF anaysis was performed using a tcl script as implemented in VMD.

$$RMSF_i = \sqrt{\frac{1}{t}\sum_{t_j=1}^{t}\left|r_i(t_j) - r_i^{ref}\right|^2} \tag{2}$$

where t is the time over which averaging was done and r_i^{ref} is the reference position of the atom i. RMSF analysis revealed the flexible regions as there were increased RMSFs values of few residues. The residues and their variation in length with elevated temperatures were presented in the Table S2. SazCA displayed a drastic change in the magnitude of RMSFs after 353 K, suggesting denaturation of the enzyme at elevated temperatures. The residues

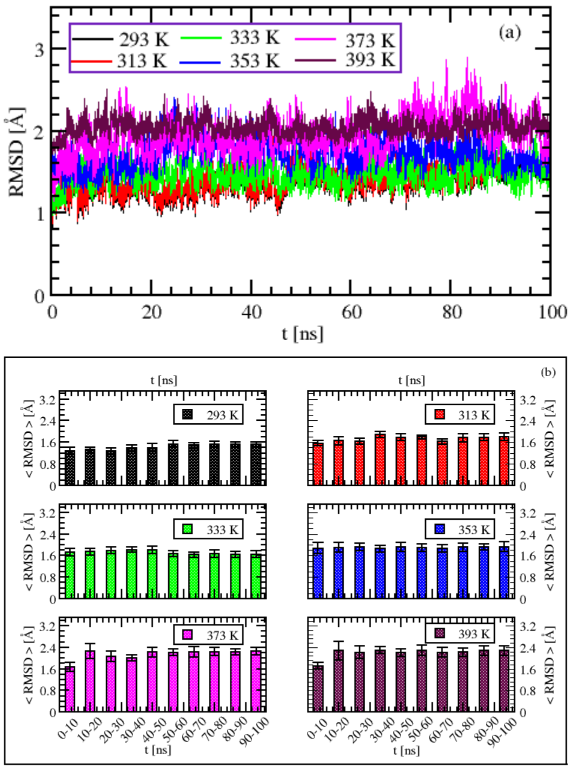

Figure S1: Evolution of average RMSD with time at different temperatures, (a) evolution of absolute RMSD and (b) variation of average RMSD, with temperature

associated with drastic increase in RMSF were indicated with bold in Table S2.

Radius of gyration (R_G) of protein as an indicator for protein structural compactness, was calculated using following equation:

$$R_G^2 = \frac{\sum_{n=1}^{n} w(i)(r(i) - \bar{r})^2}{\sum_{n=1}^{n} w(i)} \tag{3}$$

where *r(i)* is the position of atom i and $\bar{r}$ is the position of weighted center. We also calculated the time evolution of R_G as simulations progressed and this was shown in the Figure S2 below. In case of R_G also, convergence at all temperatures (293-393 K) were observed.

The measurement of solvent accessible surface area (SASA) was determined to assess the protein adaptibility and to describe the solvent accessibility. The variation in average SASA for SazCA at elevated temperatures were shown in the Figure S3.

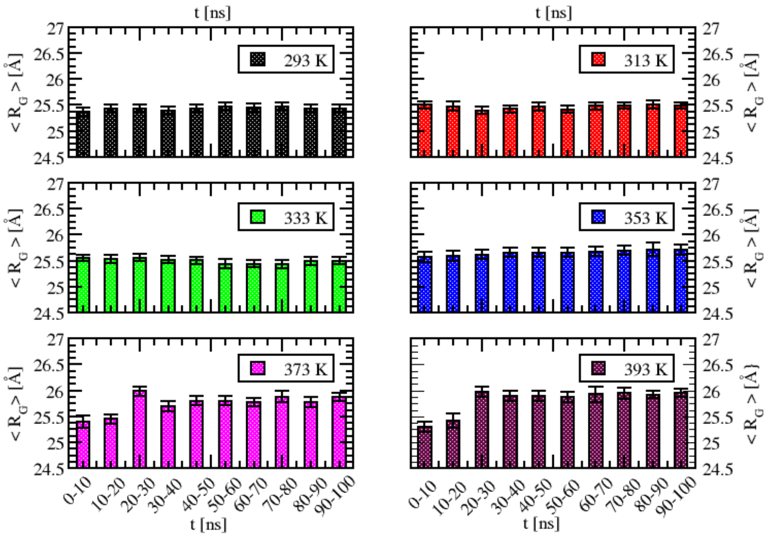

Figure S2: Evolution of average R_G of SazCA with time with different temperatures.

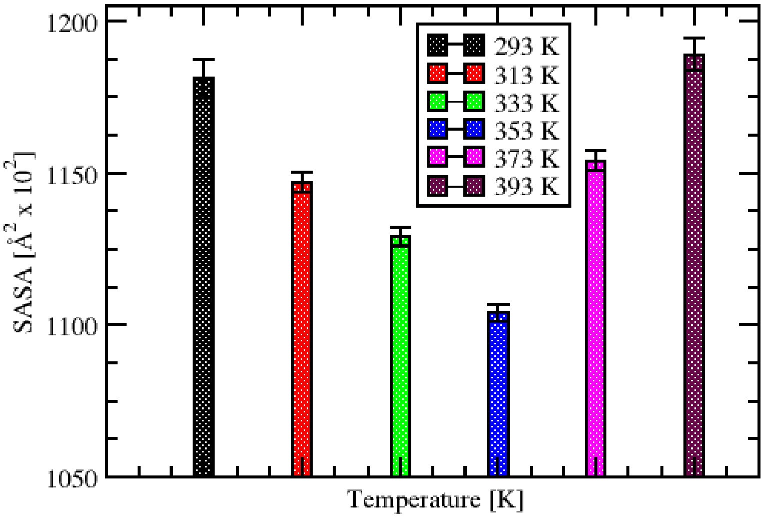

Figure S3: Average solvent accessible surface area (SASA) of SazCA at various temperatures. The error bars are the standard error of mean.

Table S1: The variation of average values of RMSD and R_G of SazCA with elevated temperatures. All the distances are in Å.

Structural parameter	Temperature (K)					
	293	313	333	353	373	393
<RMSD>	1.41 ± 0.07	1.74 ± 0.08	1.75 ± 0.08	1.94 ± 0.11	2.24 ± 0.07	2.28 ± 0.02
$<R_G>$	25.26 ± 0.08	25.49 ± 0.07	25.60 ± 0.10	25.67 ± 0.13	25.87 ± 0.13	25.90 ± 0.13

Table S2: The highly flexible amino acid residues and their variation in length with simulation temperatures. All the distances are in Å.

Residue	Temperature (K)					
	293	313	333	353	373	393
VAL98	0.75	0.79	1.74	1.52	**3.72**	**3.43**
ASN99	1.19	1.13	1.23	2.32	**4.91**	**3.71**
GLY100	1.23	1.23	2.29	2.11	**4.32**	**3.84**
LYS101	0.93	0.94	1.82	1.35	**3.32**	**3.15**
GLU145	1.02	1.11	1.07	2.15	**2.98**	**3.1**
HIS207	0.65	0.67	0.89	1.44	**2.78**	**3.2**
